# Clinical effectiveness of a standardized community-based supervised post-acute rehabilitation model after total knee arthropathy: A pilot study

**DOI:** 10.1097/ph9.0000000000000047

**Published:** 2024-11-18

**Authors:** Matthew Rong Jie Tay, Eng Chuan Neoh, Jiayen Wong, Xee Vern Tan, Chien Joo Lim, Kelvin Guoping Tan

**Affiliations:** a Department of Rehabilitation Medicine, Tan Tock Seng Hospital, Singapore; b Department of Physiotherapy, Tan Tock Seng Hospital, Singapore; c Department of Orthopedic Surgery, Woodlands Health, Singapore; d Department of Orthopedic Surgery, Tan Tock Seng Hospital, Singapore

**Keywords:** Knee, osteoarthritis, knee arthroplasty, outpatients, exercise therapy, rehabilitation center, physical therapy, delivery of health care

## Abstract

**Background:**

Hospital-based outpatient physiotherapy is the standard of care for subacute rehabilitation after total knee arthroplasty (TKA) in Singapore. This study explores the clinical effectiveness of a standardized rehabilitation model at community-based rehabilitation centers to align the appropriate utilization of tertiary and community rehabilitative resources.

**Methods:**

In this pilot study, patients who had undergone TKA were assigned to either control group (n=30) or to intervention group (n=29). The control group received usual hospital-based outpatient physiotherapy, while the intervention group received rehabilitation at a community-based rehabilitation center based on standardized institution protocol. Primary and secondary outcomes were assessed at baseline and at 3 months post TKA.

**Results:**

Baseline characteristics in both groups were not significantly different. All patients completed the study. At 3 months, there were no significant differences in the Time Up and Go test (*P*<0.853), median 30 s chair rise (*P*=0.347), knee flexion passive range of motion (*P*=0.933), knee extension passive range of motion (*P*=0.409), and presence of knee extension lag (*P*=0.360). There was a lower pain intensity in the intervention group compared with the control group (*P*=0.003).

**Conclusions:**

A community-based post-acute TKA rehabilitative model demonstrated improvements in functional outcomes, and reduced pain intensity in study participants, with these findings being similar to that of standard of care hospital-based outpatient physiotherapy. This model of care warrants further evaluation in larger clinical trials.

## Introduction

Total knee arthroplasty (TKA) is widely performed for advanced osteoarthritis of the knee after failure of conservative measures. Post-TKA rehabilitation is widely advocated and is an important aspect of recovery to manage pain and improve physical function to allow the progression of activities in patients^1,2^. Due to immediate postoperative pain limiting physical recovery, facility-based supervised physiotherapy is considered the standard of care following TKA^3–5^. The provision of a period of physiotherapy post-TKA has been shown in meta-analysis to provide at least short-term improvements in physical function^6^. However, the standard of care post-TKA physical therapy can vary highly in terms of the duration of inpatient acute physical care, rehabilitation setting to which patients are discharged after inpatient physiotherapy (eg, home-based exercises, outpatient physiotherapy, and skilled nursing facility) and rehabilitative protocols^7,8^.

With new surgical techniques and perioperative approaches, there has also been a shift in TKA care toward lower length of postoperative hospitalization and early hospital discharge to reduce perioperative morbidity^9^. However, earlier discharge to home or community settings decreases the time available for acute physical recovery, inpatient rehabilitation, and patient and family education and counseling. Although early intensive inpatient postoperative rehabilitation may enhance recovery^10,11^, patients who are discharged often require some form of supervised physical therapy post discharge^12^.

Singapore is a small island state, and post-discharge physical therapy provision for TKA patients in Singapore typically involves physiotherapy in hospital-based outpatient clinics^13^. With a constantly aging population and increasing obesity rates, the number of TKA has been projected to increase to 935,000 by 2030 in the United States alone^14^. The continued utilization of outpatient clinic services for postoperative rehabilitation poses challenges in terms of manpower, limited facility space and financial resources. In addition, although outpatient rehabilitation provides a highly equipped environment catering to patients with complex needs, postoperative TKA patients may not require such advanced levels of physiotherapy care^15^. However, the quality of post-discharge rehabilitation in the community may display a wide variation, which can lead to inappropriate therapy delivered or suboptimal outcomes, resulting in patient dissatisfaction^16–18^. Hence, in practice, postoperative rehabilitative settings are often determined by patient preferences, available resources, and longstanding referral patterns.

In Singapore, there are numerous community rehabilitation centers (CRC) situated at convenient locations, providing noncomplex and maintenance rehabilitative care for chronic conditions such as hospital-associated deconditioning and mild stroke^19^. However, such centers do not provide subacute rehabilitation for postsurgical patients due to a lack of standardized postoperative rehabilitation protocols and inadequate training. In addition, there is limited evidence on the role and outcomes of a standardized post-acute TKA rehabilitation protocol in a supervised CRC setting. Therefore, the aim of this study is to explore the differences in clinical effectiveness of a new standardized TKA community-based rehabilitation model compared with usual hospital outpatient postoperative physiotherapy in terms of patient outcomes.

## Materials and methods

### Study design

This was a pilot study conducted in Singapore from January 2021 to July 2022. The study was approved by the National Healthcare Group Domain Specific Review Board (DSRB 2019/01135). All patients provided written informed consent before participating in the study. The study was carried out in accordance with relevant guidelines and regulations of the Declaration of Helsinki. This pilot study was retrospectively registered with ClinicalTrials.gov (NCT06270446).

### Participants

All patients who had undergone a TKA were screened for eligibility.

The inclusion criteria for participants were (i) unilateral TKA; (ii) age 55 years old and above; (iii) able to engage in outpatient physiotherapy; (iv) postoperative knee flexion ≥75 degrees and knee extension ≤5 degrees; and (v) able to ambulate independently preoperatively. Exclusion criteria included patients who (i) had revision TKA or fully constrained knee arthroplasty; (ii) had knee replacement for indications other than osteoarthritis; (iii) had postoperative complications during hospital stay; (iv) were unable to participate due to cognitive impairment or language barriers; (v) had acute spinal diseases or joint, muscle or systemic diseases affecting gait; and (vi) had comorbid health conditions that would prevent active participation (eg, prior hemiplegic stroke and severe cardiorespiratory illnesses).

This pilot study was conducted to determine the preliminary estimates of the effectiveness of a community-based rehabilitation program for post-TKA patients, and hence, no a priori sample size calculation was carried out. Based on the number of previous TKA surgeries in the participating hospital, we proposed a sample size of 30 patients for each arm that was projected to be achievable in the projected study timeframe.

As with most physiotherapy interventions, it was not feasible to blind patients or therapists to treatment allocation. However, the data analysis stage was blinded.

**Figure 1:**
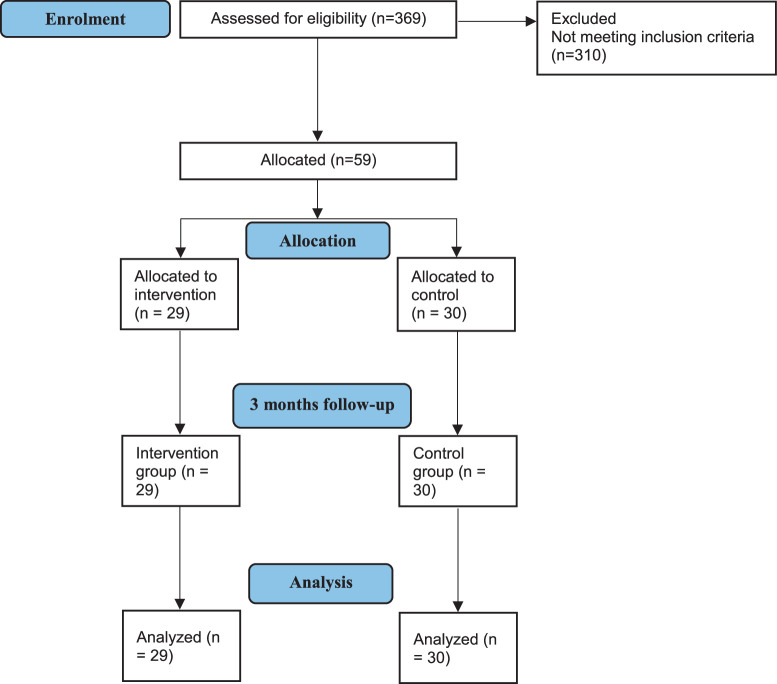
Study profile.

A total of 369 subjects were assessed for eligibility, with 310 subjects not meeting eligibility criteria. Fifty-nine patients were assigned to either the CRC (n=29) or the control group (n=30). We were unable to assign 30 patients into the CRC group due to difficulties in recruitment. Figure 1 demonstrates the flow diagram.

### Interventions

All participants in the study received usual perioperative care, and had standardized inpatient postoperative rehabilitative care based on the institution’s inpatient care protocols. All the patients are discharged 1–3 days after arthroplasty, and physiotherapy during this short inpatient stay was focused on basic mobility and knee range of motion exercises in accordance with usual hospital care, with the goal of achieving a safe hospital discharge.

Study participants were allocated to either the control group or intervention (CRC) group based on their preference. The control group received postoperative physiotherapy at a tertiary hospital outpatient physiotherapy clinic. The intervention (CRC) group received physiotherapy at a CRC based on preference and proximity. There were 4 CRCs that participated in this study: Ren Ci Day Rehab Center, St Luke’s ElderCare Day Rehab Centre, AWWA Rehab and Day Care Centre, and SPD Day Rehab Center. These CRCs were located within the regional area served by the tertiary hospital.

For both groups, participants will receive 5 physiotherapy sessions, starting from the second week post-TKA and every 2 weeks until 3 months post-TKA. Participants did not receive any physiotherapy before the second week post-TKA (apart from immediate postoperative inpatient physiotherapy, as stated previously). The post-TKA rehabilitation program was based on the pre-existing rehabilitation protocol at the tertiary hospital (Appendix 1, Supplemental Digital Content 1, http://links.lww.com/PH9/A16). Briefly, these consisted of patient education, gait retraining, range of motion exercises (eg, manual therapy and exercise therapy), strengthening exercises, and home exercises. The protocol was goal-driven, with standardized criteria for progression. Although target goals were defined, the physical therapists had flexibility in deciding how the goals could be achieved using local facilities and available equipment, with a focus on exercise-based therapy. If participants achieved the discharge criteria (Appendix 1, Supplemental Digital Content 1, http://links.lww.com/PH9/A16) during any of their physiotherapy sessions before 5 sessions were completed, they were discharged from rehabilitation. To ensure standardization of the post-TKA rehabilitation program among the various CRCs, workshops and clinical attachments were arranged for CRC physical therapists. In addition, all CRC physical therapists had to pass a clinical competency test. Site visits were conducted at the CRCs to ensure adherence to protocol. Patients in both the control and intervention groups were also provided home exercises in line with usual hospital care (Appendix 2, Supplemental Digital Content 2, http://links.lww.com/PH9/A17), although compliance was not monitored.

The CRC physical therapists were also trained to recognize potential medical issues (eg, leg swelling, delayed wound site healing, and persistent leg pain) and serious conditions (eg, severe wound infection, periprosthetic fracture, and joint injection). Patients with medical issues were discussed with a designated physical therapist in the tertiary hospital, and an early medical appointment with the orthopedic surgeon was scheduled if required. Patients with serious conditions were directed to the nearest Emergency Department.

### Outcome measures

Baseline demographic characteristics of the participants were collected. Outcome measures were assessed at the first physiotherapy session (baseline) and at the 3-month mark.

The primary outcome measures assessed were Timed up and Go (TUG) test, 30 seconds chair stand test (30CST), and numerical pain intensity.

For the TUG test, participants were asked to stand up from a standard chair with armrests, walk at a comfortable and safe pace to a 3-m marked line, walk back to the chair, and sit down. The time taken to complete this test was then recorded using a stopwatch and the average of 3 trials was calculated. The test has shown excellent inter-rater and test-retest reliability^20,21^, and excellent correlation with pain and knee-specific functional scores^22^. A longer time indicates a worse function in patients with TKA^23^.

For the 30CST, participants were asked to sit and stand as quickly and safely as possible in 30 s from a standardized chair^24^. The starting position of subjects and hand placement were standardized. The 30CST reflects lower extremity strength assessment, with a lower number indicating worse strength. It has demonstrated excellent inter-rater and test-interest reliability^25^, and excellent correlation with knee-specific functional scores^26^.

For pain intensity, patients were asked to grade the pain of the operated knee using an 11-point Numeric Rating Scale (NRS) pain score. This ranged from 0 (no pain) to 10 (worst pain imaginable)^27,28^.

Secondary outcome measures obtained were knee flexion and extension passive range of motion (PROM) and active knee extension lag. A long arm goniometer was used to measure passive knee flexion and extension ROM with the patients in a long sitting position and active knee extension lag with patients in a seated position.

Data were collected on adverse events, including wound infection, wound dehiscence, or postoperative complications. Any patient readmission within 30 days postoperation was also recorded.

### Statistical analysis

All statistical analyses were performed using IBM Statistical Package for the Social Sciences Version 26.0 (SPSS Inc.). The distribution of the continuous data was checked using skewness, kurtosis, and histograms. Continuous data were presented as mean±SD if the data were normally distributed, and presented as median (25th percentile and 75th percentile) if the data were non-normally distributed. Categorical variables were presented in frequencies and percentages.

For between-group differences, an independent *t* test was used for comparison of age, TUG test, 30 s chair stand test, numerical pain intensity, passive knee flexion ROM, length of postoperative hospitalization stay, and postoperative rehabilitation sessions as the distribution of the data were found to be normally distributed in both groups. Pearson χ2test was used for the comparison of gender, and the Fisher exact test was used for the comparison of race and preoperative ambulation status. Mann-Whitney *U* test was used for comparison of passive knee extension ROM and active quadriceps extension lag as the data were non-normally distributed. Wilcoxon signed-rank test was used for the TUG test, 30 s chair stand test, numerical pain intensity, passive knee flexion and extension ROM, and active quadriceps extension lag. All the tests were 2-sided, with statistical significance determined by *P*<0.05.

## Results

The baseline preoperative characteristics of the study participants are demonstrated in Table 1. There were no statistically significant differences between the control and intervention groups in terms of baseline characteristics, length of postoperative hospitalization stay, and the number of postoperative rehabilitation sessions received.

**Table 1:** Baseline characteristics of the participants on recruitment.

	Community rehabilitation center (n=29)	Hospital physiotherapy (n=30)	*P*
Age, y, mean±SD	70.41±7.88	67.97±7.53	0.227
Sex, n (%)			0.296
Male	7 (24.1)	11 (36.7)	
Female	22 (75.9)	19 (63.3)	
Race, n (%)			>0.950
Chinese	25 (86.2)	25 (83.3)	
Indian	1 (3.4)	2 (6.7)	
Malay	3 (10.3)	3 (10.0)	
Preoperative ambulation status, n (%)			0.443
Community	24 (82.8)	27 (90.0)	
Homebound	1 (3.4)	2 (6.7)	
Limited outdoor	4 (13.8)	1 (3.3)	
Preoperative TUG test, s, mean±SD	26.34±13.34	26.51±12.57	0.965
Preoperative 30 s chair stand test, repetitions, mean±SD	8.52±2.58	7.55±3.46	0.244
Preoperative numerical pain intensity, mean±SD	3.31±2.35	3.43±2.01	0.829
Preoperative passive knee flexion ROM, degrees, mean±SD	98.66±10.97	104.47±14.87	0.094
Preoperative passive knee extension ROM, degrees, median (IQR)	5 (0, 5)	3 (0, 5)	0.673
Preoperative active quadriceps extension lag, degrees, median (IQR)	0 (0, 5)	5 (0, 5)	0.263
Length of postoperative hospitalization stay, d, mean±SD	2.76±1.41	3.33±1.21	0.098
Postoperative rehabilitation sessions, mean±SD	4.93±0.258	4.83±0.379	0.251

Values are presented as mean±SD, number (percentage), or median (interquartile range).

ROM indicates range of motion; TUG, Time Up and Go.

The mean number of rehabilitation sessions in the control group was 4.83±0.379 and 4.93±0.258 for the intervention group. All patients completed a minimum of 4 sessions.

Table 2 reports the differences in outcomes between baseline (first session) and at 3 months. For the intervention group, there were significant improvements in TUG (*P*<0.001), 30 s chair stand (*P*=0.001), numerical pain rating (*P*<0.001), passive knee flexion PROM (*P*<0.001), passive knee extension PROM (*P*=0.001), and active quadriceps extension lag (*P*=0.004). Similarly, in the control group, there were significant improvements in TUG (*P*=0.006), 30 s chair stand (*P*<0.001), numerical pain rating (*P*=0.034), passive knee flexion PROM (*P*<0.001), passive knee extension PROM (*P*=0.012), and active quadriceps extension lag (*P*=0.003).

**Table 2:** Changes in clinical outcomes from assessment (postoperative day 1) to assessment at 3 months.

Clinical outcomes	Baseline (first session)	3 mo	*P*
Community rehabilitation center (n=29)			
TUG test, s, median (IQR)	21.41 (14.94, 34.46)	12.93 (9.30, 18.72)	<0.001
30 s chair stand test, repetitions, median (IQR)	8.00 (7.00, 10.00)	12.00 (9.00, 13.00)	0.001
Numerical pain intensity, median (IQR)	3 (2, 5)	0 (0, 1)	<0.001
Passive knee flexion ROM, degrees, median (IQR)	95.0 (90.0, 110.0)	118.0 (105.0, 120.0)	<0.001
Passive knee extension ROM, degrees, median (IQR)	5.00 (0.00, 5.00)	0.00 (0.00, 1.00)	0.001
Active quadriceps extension lag, degrees, median (IQR)	0.0 (0.0, 5.0)	0.0 (0.0, 0.0)	0.004
Hospital physiotherapy (n=30)			
TUG test, s, median (IQR)	22.52 (17.15, 36.74)	14.00 (9.90, 17.35)	0.006
30 s chair stand test, repetitions, median (IQR)	8.00 (6.00, 10.00)	10.50 (8.75, 12.00)	<0.001
Numerical pain intensity, median (IQR)	3 (3, 4)	3 (0, 3)	0.034
Passive knee flexion ROM, degrees, median (IQR)	105.0 (95.0, 115.0)	115.0 (110.0, 120.0)	<0.001
Passive knee extension ROM, degrees, median (IQR)	3.00 (0.00, 5.00)	0.00 (0.00, 4.25)	0.012
Active quadriceps extension lag, degrees, median (IQR)	5.0 (0.0, 5.0)	0.0 (0.0, 2.5)	0.003

Values are presented as median (interquartile range).

ROM indicates range of motion; TUG, Time Up and Go.

With regard to primary outcomes at 3 months, the median TUG for the intervention group was 12.93 [interquartile range (IQR): 9.30–18.82] and 14.00 (IQR: 9.90–17.35) for the control group, which were not significantly different (*P*=0.853). The median 30 s chair rise for the intervention group was 12.00 (IQR: 9.00–13.00) and 10.50 (IQR: 8.75​​​​​​–​12.00) for the control group, which were also not significantly different (*P*=0.347). However, participants in the intervention group had a significantly lower median pain intensity of 0 (IQR: 0–1) compared with a median pain intensity of 3 (IQR: 0–3) in the control group (*P*=0.003).

With regard to secondary outcomes at 3 months, the knee flexion PROM for the intervention group was 118.0 (IQR: 105.0–120.0) and 115.00 (IQR: 110.0–120.0) for the control group, which were not significantly different (*P*=0.933). The knee extension PROM for the intervention group was 0 (IQR: 0–1.00) and 0 (IQR: 0–4.25) for the control group, which were also not significantly different (*P*=0.409). The number of participants who had knee extension lag in the intervention group was 0 (IQR: 0–0) and 0 (IQR: 0–2.5) for the control group, which were not significantly different (*P*=0.360) (Table 3).

**Table 3:** Clinical outcomes of the intervention group and control group.

Clinical outcomes	Community rehabilitation center (n=29)	Hospital physiotherapy (n=30)	*P*
Primary clinical outcomes			
TUG test, s, median (IQR)	12.93 (9.30, 18.72)	14.00 (9.90, 17.35)	0.853
30 s chair stand test, repetitions, median (IQR)	12.00 (9.00, 13.00)	10.50 (8.75, 12.00)	0.347
Numerical pain intensity, median (IQR)	0 (0, 1)	3 (0, 3)	0.003
Secondary clinical outcomes			
Passive knee flexion PROM, degrees, median (IQR)	118.0 (105.0, 120.0)	115.0 (110.0, 120.0)	0.933
Passive knee extension PROM, degrees, median (IQR)	0.00 (0.00, 1.00)	0.00 (0.00, 4.25)	0.409
Active quadriceps extension lag, degrees, median (IQR)	0.0 (0.0, 0.0)	0.0 (0.0, 2.5)	0.360

Values are presented as median (interquartile range).

ROM indicates range of motion; TUG, Time Up and Go.

There were no adverse events recorded for both groups. None of the patients were readmitted to hospital within 30 days postoperation.

## Discussion

The study focused on addressing the existing lack of literature on the provision of standardized post-acute TKA rehabilitation within community settings. This pilot study reports the clinical outcomes of a group of participants who received standardized community-based supervised post-TKA rehabilitation, compared with a control group who received standard hospital-based outpatient rehabilitation.

The use of a hospital-based rehabilitation program after surgery offers several benefits to patients in terms of familiarity with the patient, standardized rehabilitation protocols, and availability of advanced rehabilitative equipment. However, the increasing number of TKA from an increasingly aging population poses significant financial and manpower constraints to the traditional hospital-based postoperative rehabilitation model^29,30^. Unfortunately, there also exists a wide range of what constitutes standard practice in terms of functional goals and rehabilitative techniques for post-TKA care in community-based rehabilitative settings, which may make referring providers hesitant about referring such patients to a non-outpatient setting. In addition, surgeons may not wish to refer to community-based rehabilitative settings postoperatively due to worries about different local protocols and guidelines with regard to postoperative care and monitoring of the patient. This has led to a lack of consensus on the optimal rehabilitative setting among surgeons, physical therapists, and patients^3^. The study’s successful completion bears significant implications for the provision of post-acute TKA rehabilitation in community settings, shedding light on the practicability of CRCs in delivering post-TKA rehabilitative care, with comparable efficacy and safety without report of adverse events.

The study results indicated that there the intervention group was comparable to the control groups for most primary outcomes (ie, TUG, 30 s chair rise), as well as secondary outcomes (knee PROM and knee extension lag), with the exception of improved numerical pain intensity in the intervention group. This shows the efficacy of both rehabilitation models in terms of postoperative knee recovery and lower limb functional outcomes. There is consistent evidence that the rehabilitative setting does not affect TKA outcomes in uncomplicated patients^31^. A purportedly more intensely supervised clinic-based setting may not provide superior recovery to non–clinic-based settings, despite clinic-based programs being delivered at greater costs^32–34^. It is postulated that an approach incorporating supervised therapy, as was undertaken in this community rehabilitation model, was critical in ensuring outcomes were at least equivalent to hospital-based settings. This is supported by evidence showing that patients undergoing supervised rehabilitation had better mobility scores and self-reported outcomes compared with a home exercise-based approach with minimal supervision^35^. We also believe that a standardized postoperative approach for early mobility, motor function training, knee ROM exercises, strength training, and appropriate goals based on safety, functional tolerance, and physiological response were key for functional recovery in TKA patients^10^. Although there has been no consensus on how to use rehabilitation visits after TKA, we found that a standardized visit frequency, guided by the patient’s recovery trajectory was successful in achieving good outcomes. Careful design of physiotherapy visits can be used to improve care quality and efficiency while reducing overall costs^36^.

There are some advantages of providing post-TKA rehabilitation in community-based settings^37^. A community-based rehabilitation program enables patients to navigate the transition from acute care to post-acute community care services by providing timely access to quality physiotherapy care while also shifting noncomplex rehabilitative services to the community in a cost-effective manner^38^. Compared with hospital-based outpatient clinics, a community-based rehabilitation model offers convenience for patients. Travel and access to hospital facilities may be a substantial hurdle for patients with post-acute TKAs. Driving or taking public transit to access care can be a painful, time-consuming, or costly experience^39^. Hence, we speculate that the reduced pain intensity in the intervention group may be attributed in part to the lesser traveling time required for study participants due to the closer proximity of the CRCs compared with the hospital’s physiotherapy clinic.

### Limitations

Our study had several limitations. We did not perform randomization for patient allocation to different groups to reduce selection bias and control for confounding. Although baseline characteristics of the 2 groups were found to be similar, a follow-up randomized trial is required to eliminate confounding effects in this study from patient allocation via preference. We did not use other functional outcomes (such as Western Ontario and McMaster Universities Arthritis Index scores) or other patient-reported outcome measures due to limitations in manpower^40^. Due to the limitation of a pilot study design, we were only able to collect outcomes at 3 months, and future studies should evaluate outcomes at shorter and longer time points. We were also unable to capture the compliance and intensity of exercises that patients performed at home (eg, using an exercise log), which may have influenced their outcomes. We also did not perform a cost-effectiveness comparison between both groups. A qualitative survey of the various factors influencing patient perspectives toward community-based rehabilitation was also not carried out. As this is a pilot study, the small sample might limit the generalizability of the findings. Further studies with larger sample sizes, longer follow-up durations, and robust study designs are warranted to validate and expand upon these preliminary findings.

## Conclusions

This study provides preliminary insights into the clinical effectiveness of a novel TKA community rehabilitation model. A standardized community-based post-acute TKA rehabilitative model demonstrated improvements in functional outcomes, with no adverse events reported. These findings highlight the potential of CRCs in offering effective post-acute TKA rehabilitation, indicating the need for further investigations to ascertain the long-term benefits and sustainability of community-based rehabilitation approaches as part of the continuum of TKA perioperative care.

## CRediT author statement

M.R.J.T, E.C.N., J.W., X.V.T., C.J.L., and K.G.T.: substantial contributions to conception and design, acquisition of data or analysis and interpretation of data, drafting the article or revising it critically for important intellectual content, and final approval of the version to be published; M.T.J.T. and C.J.L.: the statistics were reviewed and verified.

## Declaration of competing interest

The authors declare that they have no financial conflict of interest with regard to the content of this report.

## Funding

This study received a grant from the Tan Tock Seng Hospital Centre for Allied Health and Pharmacy Excellence (CAPE) and the National Medical Research Council, Singapore.

## Ethics statement

The study was approved by the National Healthcare Group Domain Specific Review Board (DSRB 2019/01135). All patients provided written informed consent before participating in the study.

## Data availability statement

The data sets generated during and/or analyzed during the current study are available from the corresponding author upon reasonable request.

## Supplementary Material
