# Supplementary material for: Clinical effectiveness of a standardized community-based supervised post-acute rehabilitation model after total knee arthropathy: A pilot study
Source: J Int Soc Phys Rehabil Med. 2024 Nov 18;7(4):129–35. doi: 10.1097/ph9.0000000000000047 (PMC11654452; doi:10.1097/ph9.0000000000000047)
Supplement: Supplementary file 1 [file ph9-7-129-s001.docx]

**Appendix 1: Post Total Knee Arthroplasty Rehabilitation Protocol**

**Phase I – Immediate Post-surgical Phase**

Goals

- Reduce swelling, pain and inflammation
- Increase knee range of motion, aim 0-90 degrees
- Ensure good quadriceps activation and no lag on active straight leg raise
- Normalize gait pattern with or without walking aid
- Stairs training if necessary

Components

- Education
  - Frequent cryotherapy/medication for pain, swelling and inflammation management
  - Limb elevation and effleurage for swelling management
  - Importance of exercise to improve strength, range of motion, ambulation and reduce possibility of knee pain in future
  - Role of physiotherapy, goals for patient and number of sessions required
- Gait retraining
  - Progression/wean off walking aid
  - Ensure equal weight shift with weight shifting and stepping exercise
  - Encourage trunk, hip and knee extension during stance phase
  - Encourage knee flexion during swing phase with stepping/crossing curb
- Increase range of motion
  - Manual therapy: Passive physiological knee extension or flexion, passive accessory mobilization for tibia or patella, fat pad mobilization, scar massage around scar or over scar once healed, soft tissue massage for distal/lateral quadriceps, tensor fascia lata/iliotibial band, hamstrings, calf (proximal), popliteus
  - Exercise therapy: Active assisted knee flexion and extension in supine or prone, supine knee hangs, stretching for hamstring, calves and quadriceps, cycling e.g. pedal bike
- Increase lower limb strength
  - Quadriceps: static/isometric quadriceps, terminal knee extension
  - Proximal and distal muscles: Bridging, sit to stand, sidelying hip abduction/clamshell (if knee range sufficient), hamstring curls (prone or standing), heel raises
  - Neuromuscular electrical stimulation for quadriceps

**Phase II – Conditioning to Advanced Phase**

Goals

- Minimal pain, swelling or warmth
- Maximize range of motion, aim 0-120 degrees
- Achieve normal gait pattern without aid/ as premorbid (e.g. equal weight bearing and no excessive hip/ knee flexion during stance)
- To be able to climb stairs (4-6 inches) in alternate steps with minimal or no pain
- To be able to do modified squat with minimal or no pain
- No limitation in usual daily activities outdoor/ return to work
- Restore balance and proprioception

Components

- Continue cryotherapy for pain, swelling and inflammation management as indicated, especially if increase warmth post exercise
- Continue range of motion and gait training
- Strength training
  - Active straight leg raise, inner range quadriceps knee extension exercise and long arc quadriceps exercise
  - Step up, side step up, step down exercise
  - Wall slides, forward lunges
  - Heel raises
  - Use of resistance/gym equipment if suitable
- Proprioception exercise e.g. static standing with feet together, semi-tandem, tandem, single leg stance, uneven surface, rockerboard

**Discharge criteria**

- Active range of motion 0 to >100 degrees
- Minimal pain (Visual Analog Score of 0-3/10) during activity
- No active quadriceps extension lag
- Normal gait
- Able to climb 4-6 inch stairs with alternate step, with minimal or no pain
- Able to perform modified squat
