# Supplementary material for: Clinical effectiveness of a standardized community-based supervised post-acute rehabilitation model after total knee arthropathy: A pilot study
Source: J Int Soc Phys Rehabil Med. 2024 Nov 18;7(4):129–35. doi: 10.1097/ph9.0000000000000047 (PMC11654452; doi:10.1097/ph9.0000000000000047)
Supplement: Supplementary file 2 [file ph9-7-129-s002.docx]

**Appendix 2: Post Total Knee Arthroplasty Home Exercises**

Patients were advised to perform the following exercises 3 times a day.

1. Breathing exercises (10 repetitions)
2. Ankle pump exercises (20 repetitions)
3. Static isometric quadriceps exercises with knee in extension (5-s hold, 10 repetitions)
4. Self-assisted active knee flexion ROM exercises (5-s hold, 10 repetitions)
5. Single leg bridging (5-s hold, 10 repetitions) on the nonoperative leg)
6. Inner range quadriceps exercises (5-s hold, 10 repetitions)
7. Hip abduction active ROM exercises (10 repetitions)
